# Human lung epithelial cell A549 proteome data after treatment with titanium dioxide and carbon black

**DOI:** 10.1016/j.dib.2016.06.013

**Published:** 2016-06-21

**Authors:** Ngoc Q. Vuong, Patrick Goegan, Susantha Mohottalage, Dalibor Breznan, Marianne Ariganello, Andrew Williams, Fred Elisma, Subramanian Karthikeyan, Renaud Vincent, Premkumari Kumarathasan

**Affiliations:** a Inhalation Toxicology Laboratory, Environmental Health Science and Research Bureau, Health Canada, Ottawa, ON, Canada K1A 0K9; b Analytical Biochemistry and Proteomics, Environmental Health Science and Research Bureau, Health Canada, Ottawa, ON, Canada K1A 0K9; c Biostatistics Section, Population Studies Division, Environmental Health Science and Research Bureau, Health Canada, Ottawa, ON, Canada K1A 0K9; d Department of Biochemistry, Faculty of Science, University of Ottawa, Ottawa, ON, Canada K1H 8M5; e Interdisciplinary School of Health Sciences, Faculty of Health Sciences, University of Ottawa, Ottawa, ON, Canada K1H 8M5

**Keywords:** Titanium dioxide, Carbon black, A549, Proteomics, 2D-GE, MALDI-TOF-TOF-MS, Toxicity

## Abstract

Here, we have described the dataset relevant to the A549 cellular proteome changes after exposure to either titanium dioxide or carbon black particles as compared to the non-exposed controls, “*Proteomic changes in human lung epithelial cells (A549) in response to carbon black and titanium dioxide exposures*” (Vuong et al., 2016) [1]. Detailed methodologies on the separation of cellular proteins by 2D-GE and the subsequent mass spectrometry analyses using MALDI-TOF-TOF-MS are documented. Particle exposure-specific protein expression changes were measured via 2D-GE spot volume analysis. Protein identification was done by querying mass spectrometry data against SwissProt and RefSeq protein databases using Mascot search engine. Two-way ANOVA analysis data provided information on statistically significant A549 protein expression changes associated with particle exposures.

**Specifications Table**

**Table:** 

Subject area	*Biochemistry and in vitro toxicology*
More specific subject area	*Toxicology of particulate matter*
Type of data	*Tables*
How data was acquired	*2D-GE was carried out using a PROTEAN IEF cell to separate proteins based on isoelectric point in the first dimension, and proteins were separated based on molecular weight by SDS-PAGE using a Criterion™ Dodeca™ Cell. Analysis of 2D gels was conducted by PDQuest*^*TM*^*Advance V8.0.1. Protein spots were excised from 2D gels using ExQuest, an automated robotic instrument. MALDI-TOF-TOF-MS analysis of tryptic-digested peptides from 2D-GE gel spots for protein identification was done using a Bruker Autoflex III Smartbeam instrument. The mass spectral data were queried using Mascot against SwissProt and RefSeq.*
Data format	*Raw, filtered and analyzed.*
Experimental factors	*A549 human lung epithelial cell line was exposed to TiO*_*2*_*and CB at 4 different doses of 0, 60, 140 and 200 μg/cm*^*2*^*. Protein expression changes were based on Coomassie blue staining of 2D gels.*
Experimental features	*A549 cells were exposed to TiO*_*2*_*and CB for 24 h. The molecular mechanisms underpinning particles’ toxicity were examined using 2D-GE- and mass spectrometry-based proteomic analysis.*
Data source location	*Environmental Health Centre, 50 Colombine Driveway, Ottawa, Ontario, K1S-0K9, Canada*
Data accessibility	*Data are within this article*

**Value of the data**

• The 2D-GE map data set the basis for particle induced-changes in A549 proteome.
• Identification of A549 proteins permits the analysis of A549 proteome.
• Particle-induced A549 protein expression changes along with protein identification help to characterize toxicity mechanisms and related altered cellular functions.
• These data and the toxicoproteomic approach are promising in the study of toxicity of environmental particles and engineered nano-materials.

## 1 Data

There are three datasets in this article. The first is the 2D-GE protein expression dataset associated with A549 cells exposed to TiO_2_ and CB particles (Supplentary Table 1). The second dataset describes the corresponding protein identification results based on MALDI-TOF-TOF-MS analyses (Supplentary Table 2). The third dataset reveals the 2D-GE protein spots that significantly changed due to particle exposures (Supplentary Table 3).

## 2 Experimental design, materials and methods

### 2.1 Experimental design

Culture flasks (T-25 and T-75), 96-well plate and plastic cell scraper were obtained from Corning Inc. (Corning, NY). Dulbecco׳s Modified Eagle׳s Medium (DMEM) and fetal bovine serum (FBS) were purchased from HyClone (Logan, UT). Gentamicin, trifluoroacetic acid, α-cyano-4-hydroxy-cinnamic acid, Tris–HCl, NaCl, Tween-20 and Tween-80 were obtained from Sigma-Aldrich (Oakville, ON). Iodoacetamide, bis-acrylamide, ammonium persulfate, glycerol, immobilized pH gradient strips, Criterion Cassette (13.3×8.7 cm W×L), Tris/Glycine/SDS buffer, and BioSafe Coomassie Blue were purchased from Bio-Rad (Mississauga, ON). Trypsin, resazurin reduction (CellTiterBlue®) and lactate dehydrogenase (LDH) (CytoTox-96®) cytotoxicity assay kits were from Promega Corporation (Madison, WI), ATP assay kits (ViaLight™ Plus) were purchased from Lonza Corporation (Rockland, ME), and 5-bromo-2′-deoxyuridine (BrdU) cell proliferation ELISA (chemiluminescent) assay kits were obtained from Roche Diagnostics (Laval, QC). All water used was deionized/demineralized (>16 MΩ resistivity).

### 2.2 Particles preparation

TiO_2_ (SRM-154b) obtained from the National Institute of Standards and Technology (Gaithersberg, MD) was subjected to three successive washes with methanol and then phosphate buffered saline (PBS) to remove possible soluble metals and organic contaminants before use in the experiments [2]. Carbon black (Cas#1333-86-4) obtained from Cabot Corporation (Boston, MA) was used as received. Particles were resuspended at 10 mg/mL in particle buffer (0.19% NaCl and 25 μg/mL Tween-80) [3], vortexed (30 s), sonicated (20 min on ice), homogenized with a Dounce Homogenizer (25 strokes), and then heated (56 °C, 1 h). The particles were stored at −40 °C until use.

### 2.3 Cell culture and particle exposure

The A549 cell line (American Type Culture Collection - CCL-185; human, epithelial, lung carcinoma) was subcultured in DMEM supplemented with 50 μg/mL gentamicin and 10% FBS. The cells were maintained in T-75 flasks in a humidified atmosphere at 37 °C containing 5% CO_2_ and 95% air. For experiments, the cells were seeded at 1.5×10^6^ cells (T-25), 3.75×10^6^ cells (T-75) or 2.0×10^4^ cells/well (96-well plate for cytotoxicity assays) and incubated for 24 h, resulting in approximately 75% confluence prior to dosing with particles. The final volume of culture medium was 5 mL (T-25), 15 mL (T-75) or 200 µL/well (96-well plate). Solutions of particles were prepared by thawing the frozen stocks, sonicating on ice (20 min) then diluting in the culture medium to make up dosing concentrations of 0, 60, 140 and 200 μg/cm^2^. The cells were exposed to the particles by replacing the existing culture medium with the particle-containing medium, and the flasks/plates were returned to the incubator for a 24 hour exposure to particles. To harvest the exposed cells, the medium in each flask was removed and the cells were detached from the flasks using a plastic scraper. The cell suspension was collected in cell culture medium and centrifuged at 350×*g* for 5 min, and the supernatant was discarded. The cell pellet was then washed twice with PBS. The final cell pellet was aspirated dry and stored frozen at −80 °C until further use. The integrated cytotoxicity bioassay which combined endpoints of cell viability (resazurin reduction assay), cellular membrane integrity (intracellular LDH release), and energy metabolism (ATP assay) was conducted in a 96-well plate as described in our previous study [4]. The cell proliferation (BrdU incorporation) assay was performed in a separate 96-well plate.

### 2.4 Protein extraction

The cell pellets were solubilized in a protein extraction/rehydration buffer from Bio-Rad (8 M urea, 2% CHAPS, 50 mM dithiothreitol, 0.2% Biolyte 3/10), where the volume depends on the number of cells in the pellet to achieve 1–2 µg/µL, and 1×10^6^ A549 cells was experimentally estimated to yield about 200 µg of protein. The samples were vortexed (30 s), sonicated (10 min), vortexed (30 s) and centrifuged ( 15,000×*g*, 10 min). The extracted protein in the supernatant was collected, and the concentration of protein in each extract was determined immediately using the Coomassie Plus Protein assay kit (Thermo Scientific). The extracted protein samples were stored in −80 °C until use.

### 2.5 Two-dimensional gel electrophoresis (2D-GE)

2D-GE was conducted as described in our previous study [5]. Briefly, an appropriate quantity of protein was suspended in a total volume of 200 µL of extraction buffer, and applied to an immobilized pH gradient (IPG) strips (11 cm, pH3–10 or pH5–8) in a clean disposable rehydration tray and allowed to incubate for 1 h at room temperature. The IPG strip was then overlaid with mineral oil and allowed to continue incubating overnight (16–20 h). The IPG strip was then moved to an isoelectric focussing tray, overlaid with mineral oil and subjected to isoelectric focussing using a PROTEAN IEF cell (BioRad). The focussing conditions were as follows: stage 1: linear ramp to 250 V for 20 min; stage 2: linear ramp to 8000 V for 2.5 h; stage 3: rapid ramp for 20,000 V h. The strip was then stored at −80 °C until use. The focused IPG strip was thawed and gently agitated for 10 min in equilibration buffer 1 (6 M urea, 2% SDS, 375 mM Tris–HCl, 20% glycerol, 130 mM dithiothreitol, 0.001% bromophenol blue). Then each strip was gently agitated for another 10 min in equilibration buffer 2 (6 M urea, 2% SDS, 375 mM Tris–HCl, 20% glycerol, 135 mM iodoacetamide, 0.001% bromophenol blue). The strip was then placed on a 12% SDS-PAGE gel casted in a 1.0 mm thick Criterion Cassette (13.3×8.7 cm W×L) and subjected to electrophoresis at 200 V for 65 min. Following electrophoresis, the gel was removed from the Criterion Cassette, washed for 30 min in water, stained in BioSafe Coomassie Blue (Bio-Rad) overnight (16–20 h), destained twice in water, and then imaged with a standard scanner.

To overcome the typical warping and distortion issues from gel to gel near the extremities of the pH and the molecular weight range, a common area (Fig. 2 [1]; pH 5.1−7.8 and 20-100 kDa) that clearly shows the protein spots across all experimental gels was selected to assess the proteome differences among the treatments. The protein spots within the gels were matched and quantified with PDQuest™ Advance V8.0.1 (Bio-Rad), where spot volume was quantified using the available “Local regression model (LOESS)” algorithm in PDQuest. The reported spot volume for each protein was used to compare its level of expression across the treatments. Three gels representing three biological repeats were generated for each group in this experiment to assess the particle-induced changes in the proteome of A549 cells.

### 2.6 In-gel digest, preparing protein spots for identification

To identify the protein in each spot of interest, a large set of preparative gels (10–12 gels) were prepared with 175 µg of protein/gel as described above. The gels were then stained with Biosafe Coomassie blue and imaged. The spots in preparative gels were then aligned and matched to the experimental gels using PDQuest. The protein spots were then excised from the preparative gels with an automated spot cutter equipped with a 1.5 mm cutting head (ExQuest from Bio-Rad). The excised gels corresponding to the same protein spot from different preparative gels were pooled into the same tube for maximum protein yield. The excised gels were then subjected to in-gel tryptic digest as described in our previous study [5]. Briefly, the gel spots were destained and then subjected to a 16 h digestion by trypsin (pH=7) at 37 °C. All the digested samples were evaporated under a gentle stream of N_2_ and were stored at −80 °C until further use.

### 2.7 Matrix-assisted laser desorption/ionization time-of-flight mass spectrometry (MALDI-TOF-TOF-MS/MS)

Each sample was reconstituted in 5 to 20 μL of 30% acetonitrile (ACN) in 0.1% trifluoroacetic acid (TFA) depending on the spot volume and was spotted (1.5 μL) on an AnchorChip target plate (600/384, Bruker Daltonics Ltd, Bremen, Germany) together with 1.5 μL of freshly prepared α-cyano-4-hydroxy-cinnamic acid (5 μg/μL in 50% ACN in 0.1% TFA). The spotted sample/matrix was dried under vacuum for at least 2 h. Each dried sample/matrix was washed with 2.5 μL of cold 0.1% TFA and briefly dried under vacuum. Each sample was analyzed by MALDI-TOF-TOF-MS using an automated analysis option (Bruker Daltonics, Bremen, Germany). In brief, MS scan of each spot was done to obtain the peptide mass fingerprint (PMF). Six major analyte peaks from the PMF spectrum were subjected to tandem MS (MS/MS) analysis in the “voltage lift mode”. The mass spectral information was matched against the Swiss-Prot and RefSeq databases using the Mascot search engine (Matrix Sciences) for protein identification. In the case that more than 1 protein was identified per spot, we attributed the protein with the highest score to such spot.

### 2.8 Statistical analysis

Two-way analysis of variance (ANOVA) was performed on 2D-GE data with treatment and dose as factors. When the assumption of equal variance and normal distribution were not met, the data were rank transformed. A protein spot is considered as significant if *p*<0.05. Particle treatment-related protein expression changes were normalized to the corresponding controls to obtain unadjusted fold-change values.
