# Supplementary material for: Human lung epithelial cell A549 proteome data after treatment with titanium dioxide and carbon black
Source: Data Brief. 2016 Jun 21;8:687–91. doi: 10.1016/j.dib.2016.06.013 (PMC4949731; doi:10.1016/j.dib.2016.06.013)
Supplement: Supplementary file 1 — Transparency document [file mmc1.docx]

**CONFLICT OF INTEREST**

All authors declare no conflict of interest.
